# The Smith County Medical Society, Organized in Tyler

**Published:** 1902-05

**Authors:** 


### The Smith County Medical Society, Organized in Tyler.

A large number of physicians of Smith county met in Tyler, Texas, March 24th, for the purpose of organizing a county medical society, the object of which is to elevate the standard of medicine and prevent quackery.

The society effected a permanent organization and will hold monthly sessions in Tyler, beginning April 14th, at which time a program will be carried out and medical subjects discussed.

The following officers were duly elected: President, Dr. T. J. Bell, of Tyler; Vice-President, Dr. W. S. Lacey, of Troupe; Secretary and Treasurer, Dr. Albert Woldert, of Tyler.

